# The effects of two different doses of ultraviolet-A light exposure on nitric oxide metabolites and cardiorespiratory outcomes

**DOI:** 10.1007/s00421-018-3835-x

**Published:** 2018-03-07

**Authors:** Chris Monaghan, Luke C. McIlvenna, Luke Liddle, Mia Burleigh, Richard B. Weller, Bernadette O. Fernandez, Martin Feelisch, David J. Muggeridge, Chris Easton

**Affiliations:** 1 Institute for Clinical Exercise and Health Science, University of the West of Scotland, Almada Street, Hamilton, ML3 0JB UK; 2 Institute of Sport, Exercise and Active Living, Victoria University, Melbourne, Australia; 3 Department of Dermatology, University of Edinburgh, Edinburgh, UK; 4 Clinical and Experimental Sciences, Faculty of Medicine, NIHR Southampton Biomedical Research Centre, University of Southampton and University Hospital Southampton NHS Foundation Trust, Southampton, UK; 5 Physical Activity for Health Group, School of Psychological Science and Health, University of Strathclyde, Glasgow, UK

**Keywords:** Sunlight, Nitrate, Nitrite, Blood pressure, Metabolic rate

## Abstract

**Purpose:**

The present study investigated different doses of ultraviolet-A (UV-A) light on plasma nitric oxide metabolites and cardiorespiratory variables.

**Methods:**

Ten healthy male participants completed three experimental conditions, 7 days apart. Participants were exposed to no light (CON); 10 J cm^2^ (15 min) of UV-A light (UVA10) and 20 J cm^2^ (30 min) of UV-A light (UVA20) in a randomized order. Plasma nitrite [NO_2_^−^] and nitrate [NO_3_^−^] concentrations, blood pressure (BP), and heart rate (HR) were recorded before, immediately after exposure and 30 min post-exposure. Whole body oxygen utilization (${{\dot{V}}}{\rm O}_{2}$), resting metabolic rate (RMR) and skin temperature were recorded continuously.

**Results:**

None of the measured parameters changed significantly during CON (all *P* > 0.05). ${{\dot{V}}}{\rm O}_{2}$ and RMR were significantly reduced immediately after UVA10 (*P* < 0.05) despite no change in plasma [NO_2_^−^] (*P* > 0.05). Immediately after exposure to UVA20, plasma [NO_2_^−^] was higher (*P* = 0.014) and ${{\dot{V}}}{\rm O}_{2}$ and RMR tended to be lower compared to baseline (*P* = 0.06). There were no differences in [NO_2_^−^] or ${{\dot{V}}}{\rm O}_{2}$ at the 30 min time point in any condition. UV-A exposure did not alter systolic BP, diastolic BP or MAP (all *P* > 0.05). UV-A light did not alter plasma [NO_3_^−^] at any time point (all *P* > 0.05).

**Conclusions:**

This study demonstrates that a UV-A dose of 20 J cm^2^ is necessary to increase plasma [NO_2_^−^] although a smaller dose is capable of reducing ${{\dot{V}}}{\rm O}_{2}$ and RMR at rest. Exposure to UV-A did not significantly reduce BP in this cohort of healthy adults. These data suggest that exposure to sunlight has a meaningful acute impact on metabolic function.

## Introduction

Nitric oxide (NO) is produced by NO synthases (NOS) from the oxidation of l-arginine and acts as a multifunctional signaling molecule that regulates a number of key biological processes including neuronal signaling, immune function, mitochondrial respiration, and vascular tone (Carr and Ferguson 1990; Moncada and Higgs 1991; Stamler et al. 1994). NO is rapidly oxidized within the human body to nitrite (NO_2_^−^) and nitrate (NO_3_^−^), with the latter accounting for the majority of NO-derived compounds within the body. Recent evidence suggests that NO_3_^−^ and NO_2_^−^ are important reservoirs of NO that act independently of NOS activity (Lundberg et al. 2009, 2015). Although NO_3_^−^ is considered to be physiologically inert, it can be reduced to biologically active NO_2_^−^ by bacteria in the oral cavity (Duncan et al. 1995) and gut (Tiso and Schechter 2015) or reduced by xanthine oxidoreductases (XOR) (Lundberg et al. 2009). NO_2_^−^ can initiate physiological effects or be reduced further to NO under hypoxic (Castello et al. 2006) and acidic (Modin et al. 2001) conditions.

Several studies have demonstrated that increasing plasma levels of NO_3_^−^ and NO_2_^−^ through dietary NO_3_^−^ ingestion and via sunlight exposure can reduce blood pressure (BP) (Larsen et al. 2006; Webb et al. 2008; Opländer et al. 2009; Kapil et al. 2010; Vanhatalo et al. 2010; Siervo et al. 2013; Wylie et al. 2013; Muggeridge et al. 2015; McIlvenna et al. 2017). Increasing the circulating concentration of NO metabolites has also been shown to elicit other cardiovascular and metabolic effects, including the reduction of resting oxygen utilization (${{\dot{V}}}{\rm O}_{2}$) (Larsen et al. 2014; Whitfield et al. 2016). Intriguingly, both dermis and epidermis contain NO_2_^−^ at concentrations substantially greater than those in blood (Paunel et al. 2005). Exposing the skin to light in the UV-A wavelength range (315–400 nm) leads to photodecomposition of these NO derivatives and release of NO into the circulation with consequent biological effects (Paunel et al. 2005; Mowbray et al. 2009; Suschek et al. 2010). UV-A induced NO production has been shown to be independent of NOS, temperature, and vitamin D status (Liu et al. 2014). Given that basal plasma [NO_2_^−^] is suggested to be a marker of endothelial function (Kleinbongard et al. 2006), whereby low levels correlate with increased cardiovascular risk (Allen et al. 2009), UV-A induced NO production could conceivably modulate cardiovascular homeostasis.

Despite the potential health significance of these observations, little is known about the minimal dose of UV-A exposure required to alter circulating NO levels. Whole body UV-A exposure of 20 J cm^2^ (equivalent to ~ 30 min of Mediterranean summer sunlight) has been consistently shown to mobilize NO_2_^−^ from the skin into the plasma (Liu et al. 2014) and reduce systemic BP (Opländer et al. 2009; Liu et al. 2014; Muggeridge et al. 2015). To the best of our knowledge, this has been the only dose of UV-A used in human volunteer studies related to the mobilization of NO from skin. However, it has been shown in vitro that UV-A light of 9 J cm^2^ causes a dose-dependent increase in NO production in isolated keratinocytes without causing DNA damage (Holliman et al. 2017). This may be important as current public health messages favor sun avoidance due to the established carcinogenic effects of habitual UV exposure (Kennedy et al. 2003; Rigel 2008). Given that exposure to sunlight may impact positively on markers of cardiovascular health, further research is warranted to determine the minimum dose of UV-A exposure to elicit these effects.

Therefore, the primary aim of the present study was to compare the effects of two different doses of UV-A light on plasma [NO_2_^−^]. Additional aims were to examine the effects of the same doses of UV-A light on [NO_3_^−^], BP, resting ${{\dot{V}}}{\rm O}_{2}$ and RMR. We hypothesized that UV-A light would increase plasma NO derivatives and reduce BP and resting ${{\dot{V}}}{\rm O}_{2}$ in a dose-dependent manner.

## Methods

### Participants

Ten healthy males (age 28 ± 5 years, stature 180 ± 9 cm, body mass 80.8 ± 11.0 kg) volunteered to participate in the study following ethical approval by the School of Science and Sport Ethics Committee at the University of the West of Scotland. Participants self-determined their skin types via the Fitzpatrick Skin Questionnaire (Fitzpatrick 1988) as skin type 2 (*n* = 1) and skin type 3 (*n* = 9). Written informed consent was obtained from all participants prior to the commencement of the study. All participants were non-smokers, apparently healthy, were not regular users of anti-bacterial mouthwash, and reported no use of medication. Prior to each trial, participants were instructed to avoid prolonged sunlight exposure and caffeine on the morning of each visit and to avoid NO_3_^−^ rich foods (such as beetroot and lettuce), high intensity exercise, and alcohol consumption within 48 h of each trial.

### Experimental design

Each participant attended the laboratory on three separate occasions and was exposed to either no light (control, CON), 10 J cm^2^ of UV-A light (UVA10), or 20 J cm^2^ of UV-A light (UVA20), in a randomized counter-balanced order. Each condition was separated by 7 days and performed at the same time of day for each participant. Trials were performed in Scotland at 55.78°N latitude between July and December. Each trial was conducted in the morning (before 11 a.m.) after an overnight fast and following the consumption of ~ 500 mL of bottled water upon awakening. Dietary and exercise habits were self-recorded via a 48 h recall at the beginning of visit one and repeated on each visit thereafter. Compliance to these factors was determined at the beginning of each visit.

### UV-A exposure

Whole body UV-A exposure was delivered using a commercially available UV-A light therapy system (Waldmann, UV302 L, Germany). UV-A light occurs naturally in the 315–400 nm wavelength band, and the present system emitted light at wavelengths between 315 and 351 nm. In all conditions, participants lay supine on a medical plinth for the duration of the experiment and wore shorts and protective glasses. Participants were instructed to close their eyes for the duration of the light exposures. During CON, the UV-A system remained off. The trials were conducted in a temperature-controlled laboratory (22 ± 1.3 °C). The dose of UV-A was automatically calculated based on exposure time and calibrated irradiance at a distance of 21 cm from the abdominal skin. Exposure time was 15 min in UVA10 and 30 min in UVA20. Calibration of the UV-A light source was conducted as per the manufacturer’s instructions.

### Experimental procedures

On arrival, stature and body mass were recorded following the bladder void. Upon lying supine for a period of 30 min prior to baseline measurements, an intravenous catheter was inserted into the antecubital vein of each participant for collection of venous blood samples. In the UVA10 condition, participants lay supine for an additional 15 min prior to baseline measurements to time-match the duration of the experiment across all three conditions. Skin temperature was continuously monitored (Squirrel SQ2022, Cambridge, England) via thermistors placed at four anatomical sites (triceps, chest, quadriceps, and calf). Participants were fitted with a heart rate (HR) monitor (Polar Electro, Oy, Finland), which was continuously monitored by telemetry. Resting ${{\dot{V}}}{\rm O}_{2}$ was measured via indirect calorimetry using breath-by-breath analysis (Medgraphics, Milan, Italy). The volume measurement of the system was calibrated prior to each trial using a 3-L syringe as per the manufacturers’ instructions. Gas analyser calibration was performed with two gases of known concentrations (calibration gas: 5% carbon dioxide, 12% oxygen and nitrogen balance and reference gas: 21% oxygen and nitrogen balance) (Air Liquide Healthcare, Belgium). Resting ${{\dot{V}}}{\rm O}_{2}$ data were taken as an average for 20 min at baseline, during treatment, and for 30 min post-exposure. Upper and lower limits of agreement were calculated for resting ${{\dot{V}}}{\rm O}_{2}$, and data points exceeding two standard deviations from the mean were excluded from analysis. Resting metabolic rate (RMR) was estimated using the following equation: RMR = ${{\dot{V}}}{\rm O}_{2}$ (L/min^−1^) × calorific value. The calorific values were calculated from the respiratory exchange ratio using data from Lusk (1923). Brachial BP was measured in triplicate with 1 min between measures at baseline, immediately after light exposure (0 min), and 30 min after exposure using a manual stethoscope and sphygmomanometer (Accoson, London, UK), with the pressure cuff placed at the upper part of the non-dominant arm positioned at the level of the right atrium. Mean values were used for analysis. Mean arterial BP (MAP) was calculated using the following equation: MAP = [(2 × diastolic pressure + systolic pressure)/3]. Following each BP measure, 8 mL of venous blood was collected from the opposite arm at baseline, 0 min, and 30 min post-exposure. Blood was collected in vacutainers containing EDTA and immediately centrifuged at 4000 rpm at 4 °C for 10 min (Harrier 18/80, MSE, UK). Plasma was extracted and immediately stored at − 80 °C for later analysis of [NO_3_^−^] and [NO_2_^−^]. Intravenous lines were flushed with 2 mL of 0.9% saline solution immediately following each blood draw.

### Analysis of plasma NO metabolites

Gas-phase chemiluminescence was used to determine plasma [NO_3_^−^] and [NO_2_^−^], as previously described (Pinder et al. 2008; Muggeridge et al. 2014; McIlvenna et al. 2017). Briefly, following the creation of a standard curve at several concentrations (0–1000 nM for NO_2_^−^) and (0–100 µM for NO_3_^−^), samples were thawed in a water bath at 37 °C for 3 min. Plasma samples were injected in duplicate into a customized enclosed purge vessel containing the respective reagent mixture. For the determination of NO_2_^−^, a reagent containing 1% sodium iodide in 4 mL of glacial acetic acid kept at 50 °C was used to reduce NO_2_^−^ to NO.

Before the determination of NO_3_^−^, plasma samples were deproteinized using zinc sulfate (ZnSO_4_) and sodium hydroxide (NaOH). Samples were made up in 1 mL aliquots (200 µL plasma: 400 µL ZnSO_4_ (10% w/v) and 400 µL NaOH solution (200 µL de-ionized water: 200 µL 1 M NaOH) and vortexed for 30 s. Samples were then centrifuged at 4000 rpm for 5 min and supernatants were used for NO_3_^−^ analysis. For the determination of NO_3_^−^, a reagent containing 32 mg of vanadium trichloride (VCl_3_), 4 mL of 1M hydrochloric acid, and 500 µL of de-ionized water was used in a sealed purge vessel maintained at 95 °C. NO was quantified using an NO analyzer (Sievers NOA 280; Analytix, UK). The area under the curve (AUC) was then used to determine concentrations by plotting the standard curve and dividing the AUC of each sample by the gradient of the slope. The co-efficient of variance (COV) for both plasma [NO_2_^−^] and [NO_3_^−^] was better than 5%.

### Data analysis

The distribution of the data was tested using the Shapiro–Wilk test. A two-way repeated-measures ANOVA was used to examine the differences between ‘condition’, ‘time’ and the ‘condition × time’ interaction for all variables. Post hoc analysis of the significant main effects was conducted using paired *t* tests and adjusted for multiple comparisons using the Bonferroni correction. Statistical significance was accepted at *P* < 0.05. The inclusion of 95% CI for mean differences is presented with *P* values and effect sizes (Cohen’s *D*), when appropriate. Effect sizes were interpreted as: small effect > 0.2; medium effect > 0.5; large effect > 0.8. Data were analyzed using SPSS (version 22.0) and Graph Pad Prism (version 7.02). Data in the text are presented as mean ± standard deviation. Data in figures are presented as group delta (Δ) ± standard error of the mean (SEM) relative to pre-treatment baselines.

### Results

#### NO metabolites

**Fig. 1:**
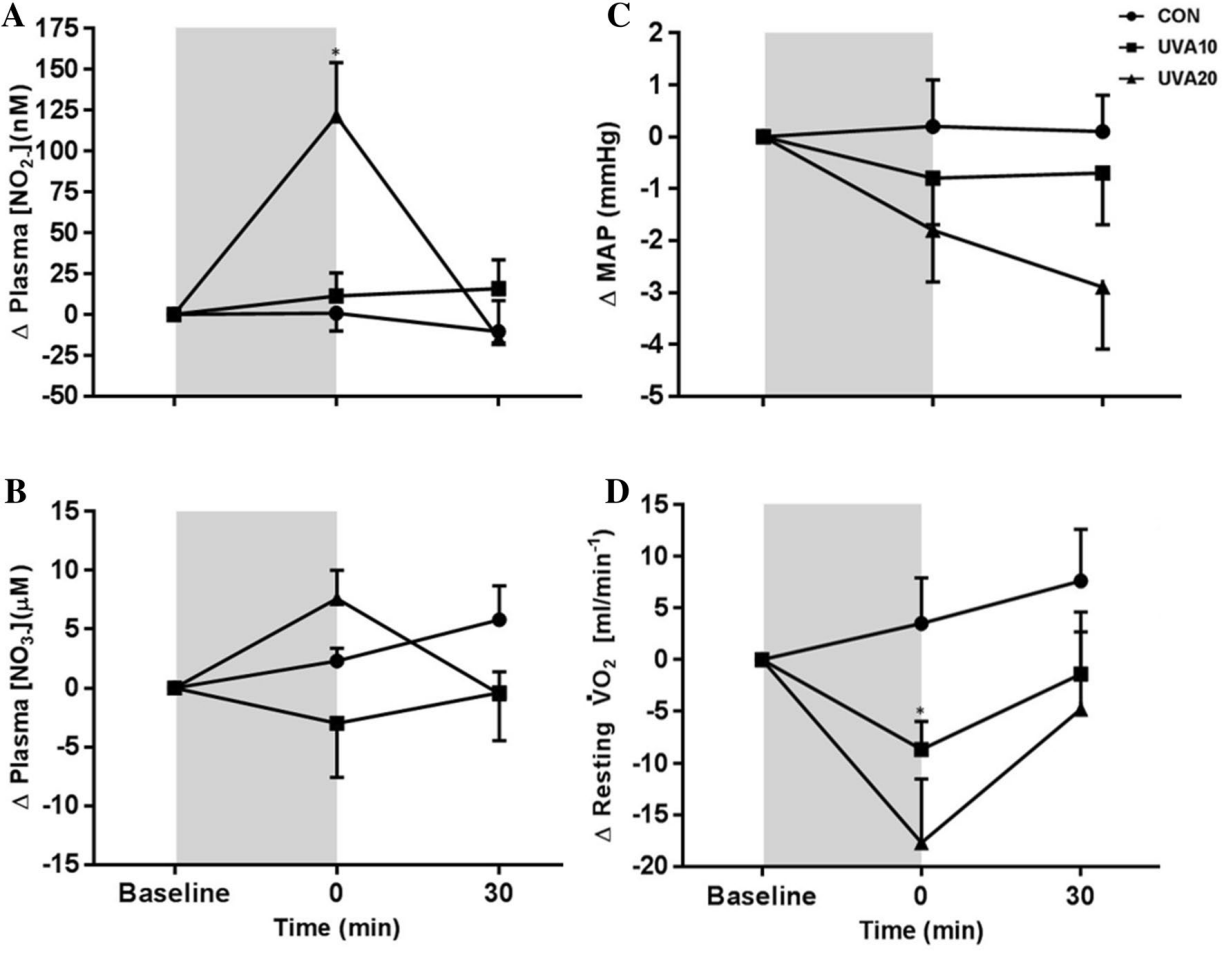
**a** Plasma [NO_2_^−^]; **b** plasma [NO_3_^−^]; **c** mean arterial pressure; and **d** resting oxygen utilization (${{\dot{V}}}{\rm O}_{2}$), **d** following no exposure [CON (circles)], 10 J cm^2^ [UVA10 (squares)], and 20 J cm^2^ [UVA20 (triangles)] of UV-A light. Data are presented as the group delta mean ± SEM. Shaded area signifies the period of UV-A light exposure in UVA10 and UVA20. *Denotes significant increase in plasma NO_2_^−^ compared to baseline

Plasma [NO_2_^−^] changes are shown in Fig. 1a. Plasma [NO_2_^−^] was not different between the three conditions at baseline (CON 109 ± 54 nM, UVA10 108 ± 55 nM, UVA20 162 ± 75 nM, *P* = 0.18). There was a significant main effect of ‘condition’ (*P* = 0.004), ‘time’ (*P* = 0.001) and ‘condition × time’ interaction (*P* < 0.001) on plasma [NO_2_^−^]. Following UVA20, plasma [NO_2_^−^] was increased from baseline at the 0 min time point (95% CI 25.7–216.9; *P* = 0.014; *d* = 1) but was not different at 30 min (*P* = 1.0; *d* = 0.2). Plasma [NO_2_^−^] did not differ from baseline at the 0 min or the 30 min time points in both the CON and UVA10 trials (all *P* > 0.4, *d* < 0.3).

Changes in plasma [NO_3_^−^] are shown in Fig. 1b. Plasma [NO_3_^−^] was different between the three conditions at baseline (CON 45.6 ± 16.2 µM, UVA10 55.1 ± 21.9 µM, UVA20 39.5 ± 16.8 µM, *P* = 0.04). Post hoc comparisons revealed baseline plasma [NO_3_^−^] was higher in UVA10 compared to UVA20 (95% CI 0.4–31; *P* = 0.05; *d* = 1), but there was no difference between CON and UVA10 (*P* = 0.4) or CON and UVA20 (*P* = 0.9). There was a significant main effect for ‘condition’ (*P* = 0.013) on plasma [NO_3_^−^]. Overall, plasma [NO_3_^−^] was significantly higher in UVA10 when compared to UVA20 (95% CI 2.5–25.6; *P* = 0.02; *d* = 1), but was not different to CON (*P* = 0.7). There was no significant main effect for ‘time’ (*P* = 0.7) or ‘condition × time’ interaction (*P* = 0.109) for plasma [NO_3_^−^].

#### Blood pressure, heart rate and skin temperature

There was no significant ‘condition x time’ interaction for systolic BP (SBP) (*P* = 0.91; Table 1), diastolic BP (DBP) (*P* = 0.19; Table 1) or MAP (*P* = 0.27; Fig. 1c; Table 1). There was no difference in BP variables at baseline *(P* > 0.5; Table 1). Although no significant difference was observed in BP, effect sizes indicate that following UVA20, DBP reduced to a small extent immediately following (*P* = 0.3; *d* = 0.3) and 30 min (*P* = 0.15; *d* = 0.3) post-exposure. After UVA20, there was a small reduction in MAP immediately following exposure (*P* = 0.3; *d* = 0.3) and a moderate decrease 30 min after exposure (*P* = 0.1; *d* = 0.5) when compared to baseline (Table 1). Skin temperature and HR data are shown in Table 1. There was no significant main effect of ‘condition x time’ for HR (*P* = 0.5). There was a trend towards a ‘condition × time’ interaction for skin temperature (*P* = 0.06). Effect sizes show a small increase in skin temperature immediately following UVA20 (*d* = 0.4), but no difference between any other time points (*d* < 0.2).

**Table 1:** Blood pressure, skin temperature and heart rate variables in each condition pre, immediately after cessation (0 min) and half an hour following UV-A light exposure

Condition and variable	Baseline	0 min	30 min
CON			
Systolic BP (mmHg)	116 ± 2	116 ± 3	115 ± 3
Diastolic BP (mmHg)	70 ± 2	70 ± 3	71 ± 3
MAP (mmHg)	85 ± 2	85 ± 3	85 ± 3
Heart rate (beats min^−1^)	60 ± 3	60 ± 3	58 ± 3
Skin temperature (^o^C)	32 ± 0	32 ± 0	32 ± 0
UVA10			
Systolic BP (mmHg)	115 ± 1	115 ± 1	115 ± 1
Diastolic BP (mmHg)	71 ± 2	70 ± 2	70 ± 2
MAP (mmHg)	86 ± 1	85 ± 2	85 ± 2
Heart rate (beats min^−1^)	58 ± 2	59 ± 3	55 ± 2
Skin temperature (^o^C)	32 ± 0	33 ± 0	32 ± 0
UVA20			
Systolic BP (mmHg)	115 ± 1	113 ± 2	113 ± 2
Diastolic BP (mmHg)	69 ± 2	67 ± 3^a^	67 ± 2^a^
MAP (mmHg)	85 ± 2	83 ± 2^a^	82 ± 2^b^
Heart rate (beats min^−1^)	58 ± 3	58 ± 2	55 ± 3^b^
Skin temperature (^o^C)	33 ± 0	34 ± 0^a^	33 ± 0

Data are presented as mean ± SEM

^a^Denotes a small effect size change compared to baseline

^b^Denotes a moderate effect size change compared to baseline (Cohen’s *D*)

#### Resting ${{\dot{V}}}{\rm O}_{2}$ and metabolic rate

There was a significant ‘condition × time’ effect on resting ${{\dot{V}}}{\rm O}_{2}$ (*P* = 0.047; Fig. 1d). Resting ${{\dot{V}}}{\rm O}_{2}$ was not different between the three conditions at baseline (CON 273 ± 37 mL/min^−1^, UVA10 267 ± 32 mL/min^−1^, UVA20 269 ± 37 mL/min^−1^; *P* = 0.8). Following UVA20, there was a trend for a reduction in resting ${{\dot{V}}}{\rm O}_{2}$ at 0 min compared to the baseline (95% CI − 0.759 to 36.1, *P* = 0.061; *d* = 0.4), but values were not different at 30 min (*P* = 1; *d* < 0.2). Following UVA10, resting ${{\dot{V}}}{\rm O}_{2}$ was significantly reduced at 0 min (95% CI 0.837–16.3; *P* = 0.03; *d* = 0.3) but did not differ from baseline 30 min after exposure (*P* = 1; *d* < 0.2). In CON, resting ${{\dot{V}}}{\rm O}_{2}$ did not change at 0 or 30 min (both *P* = 1; *d* < 0.2).

There was a significant ‘condition x time’ effect on resting RMR (*P* = 0.041). RMR was not different between the three conditions at baseline (CON 1917 ± 260 kcal/day^−1^, UVA10 1860 ± 223 kcal/day^−1^, UVA20 1883 ± 259 kcal/day^−1^; *P* = 0.5). Following UVA20, there was a trend for a reduction in RMR at 0 min compared to the baseline (Δ129 ± 42 kcal/day^−1^, 95% CI − 5 to 453, *P* = 0.056; *d* = 0.4), but values were not different at 30 min (*P* = 0.91; *d* < 0.2). Following UVA10, RMR was significantly reduced at 0 min (Δ57 ± 14 kcal/day^−1^, 95% CI 6–114; *P* = 0.04; *d* = 0.3) but did not differ from baseline 30 min after exposure (*P* = 1; *d* < 0.2). In CON, resting ${{\dot{V}}}{\rm O}_{2}$ did not change at 0 or 30 min (both *P* = 1; *d* < 0.2).

### Discussion

Exposure to 20 J cm^2^ of UV-A light, a dose equivalent to approximately 30 min of Mediterranean summer sunlight (Diffey 2002), has previously been shown to increase plasma [NO_2_^−^] and lead to a sustained reduction in BP (Opländer et al. 2009; Liu et al. 2014). The present study explored the effects of different doses of UV-A exposure and the effects on circulating NO metabolites, BP, and resting ${{\dot{V}}}{\rm O}_{2}$. The principal findings were that 20 J cm^2^ of UV-A exposure resulted in a brief, but significant increase in plasma [NO_2_^−^] whereas 10 J cm^2^ was insufficient to alter the concentration of this NO metabolite. In contrast to previous findings, exposure to UV-A light in either dose did not alter BP. However, we here demonstrate for the first time that exposure to UV-A light reduces resting ${{\dot{V}}}{\rm O}_{2}$ and RMR. While these data suggest that a minimum dose of 20 J cm^2^ is necessary to augment plasma NO availability, further work is required to better understand the therapeutic effects of UV-A light on cardiovascular and metabolic health.

#### Dose-dependent effects of UV-A light on NO metabolites

The observed increase in plasma [NO_2_^−^] following 20 J cm^2^ but not 10 J cm^2^ of UV-A light supports our original hypothesis that the UV-induced release of NO metabolites from the skin is dose dependent. The higher dose of UV-A light is proportional to approximately 30 min of Mediterranean summer sunlight (Liu et al. 2014). We demonstrate that 20 J cm^2^ of UV-A light increased plasma [NO_2_^−^] by 75% immediately following cessation of exposure, which is higher than the proportional increases of 45% (Opländer et al. 2009) and 40% (Liu et al. 2014) that have been previously reported. On the other hand, the absolute increase in plasma [NO_2_^−^] was higher (~ 200 nM) in the study by Liu and colleagues (2014) than in the present study (123 nM). Collectively, the present study and other studies in the area (Opländer et al. 2009; Liu et al. 2014; Muggeridge et al. 2015) suggest that UV-A induced NO production may alter the overall circulating pool of NO. However, it is clear that there is profound inter-individual variability of NO_2_^−^ following UV-A challenge (Opländer et al. 2009). This may be explained by recent data from Holliman and colleagues (2017) who demonstrated diverging baseline levels of skin NO content in their skin donors and a variable magnitude of NO release from isolated keratinocytes in response to UV-A exposure. Both age and body composition are known to influence the storage and release of NO metabolites from the skin (Ma et al. 2015). These variables, along with skin type and habitual sunlight exposure may help explain the divergent response to UV-A between individuals and study cohorts. The total storage of NO metabolites in the skin is likely to be important as it has recently been demonstrated in vitro that NO_2_^−^ is converted to NO upon UV-A exposure (Holliman et al. 2017).

In contrast to previous findings (Opländer et al. 2009; Liu et al. 2014), the observed increase in plasma [NO_2_^−^] immediately following exposure with 20 J cm^2^, was not sustained 30 min post-exposure. One possible explanation is that the source and delivery method for the UV-A light differed between all of these studies involving human volunteers. We speculate that the overall ‘dose’ or intensity of light may contribute to diverging NO_2_^−^ release and overall NO kinetics. Previous studies have utilized the same dose of 20 J cm^2^ of UV-A light (Opländer et al. 2009; Liu et al. 2014; Muggeridge et al. 2015), however, over different time periods and intensities. In the present study, the intensity of light was the same in UVA10 and UVA20, where exposure time was manipulated to alter the overall dose. In vitro experiments highlight the relevance of the UV-A source showing that overall NO kinetics are altered depending on the distance from the UV-A light source (Dejam et al. 2003) and potentially the specific wavelength of the light. The middle to long UV-A wavelength range of 340–400 nm has been shown to be the major contributor to overall NO production in isolated keratinocytes in response to UV-A challenge (Holliman et al. 2017). In the present study, our UV-A light source emitted its maximum intensity at 351 nm. It should be highlighted, however, that natural sunlight contains light in the UV-B wavelength which will increase production of vitamin D_3_, and potentially cause erythema and DNA damage (Marionnet et al. 2015). A key question remains, therefore, as to whether the release of NO metabolites differs in response to natural or artificial sources of light.

In contrast with previous research, plasma [NO_3_^−^] was not altered by UV-A exposure. Liu and colleagues (2014) have previously demonstrated that 20 J cm^2^ of UV-A light reduced [NO_3_^−^] by ~ 3 µM. The authors speculated that UV-A exposure may result in direct photolysis of NO_3_^−^ to yield NO. Alternatively, it was suggested that UV-A exposure may release NO_3_^−^ from skins stores which in turn would enhance the reduction of NO_3_^−^ to NO_2_^−^ via facultative bacteria in the oral cavity (Duncan et al. 1995) and gut (Tiso and Schechter 2015) or by XOR (Lundberg et al. 2009). However, the authors noted that the decline in plasma [NO_3_^−^] was over ten times the increase in [NO_2_^−^] which suggests the reduction in BP following UV-A exposure resulted from bioactivation of cutaneous rather than circulating NO stores. More recent data now suggest that photolysis of NO_3_^−^ to NO by UV-A light seems unlikely (Holliman et al. 2017). These authors demonstrated that irradiation of a solution of sodium NO_2_^−^ released NO in a dose-dependent manner. Conversely, irradiation of sodium NO_3_^−^ did not yield NO. While these contrasting data between the present research and that of Liu and colleagues (2014) are not readily explainable, it is conceivable that the aforementioned differences in UV-A delivery methods may be important. Alternatively, inter-individual variability in the release of NO metabolites following UV-A exposure (Oplander et al. 2009; Holliman et al. 2017) and the multiple biological fates of these molecules may help to explain these notable differences. While future research is warranted to elucidate the role of skin and circulating stores of NO_3_^−^, it seems likely that the consistently reported bioactivation of NO_2_^−^ is responsible for the myriad of physiological effects that occur in response to UV-A exposure.

#### Blood pressure is not altered by UV-A light

Our study demonstrated that UV-A light did not significantly alter BP. This finding contrasted our study hypothesis and previous research which has demonstrated that 20 J cm^2^ effectively reduces BP in a healthy cohort (Opländer et al. 2009; Liu et al. 2014). Nevertheless, there was a moderate reduction in MAP (*d* = 0.5) 30 min after exposure in the UV20 condition with a concomitant moderate reduction in heart rate (*d* = 0.5). While the clinical and biological significances of the small reduction in MAP (3 mmHg) in this study are unclear, a reduction of DBP by only 5 mmHg decreases risk for stroke by 34% (MacMahon et al. 1990) and any amount of BP reduction is protective against cardiovascular mortality (Lawes et al. 2004). It should be highlighted that the small sample of participants in this study were all young, healthy, and normotensive males. The most likely explanation for the absence of a significant reduction in BP was that the elevation in plasma [NO_2_^−^] was not sustained long enough to elicit a pronounced biological effect.

The experimental procedures may also have limited the extent to which UV-A light may have reduced BP. Specifically, the measurement of BP following the treatments in each condition was preceded by a 1-h period of lying supine. The consequence is that this sustained period of lying supine likely induced postural venodilation (Gemignani et al. 2008) and lowered BP prior to the experimental intervention. Indeed, DBP was 70 ± 7 mmHg and MAP was 85 ± 6 mmHg at baseline across all three conditions. This may have limited any further biologically significant reductions in BP following UV-A exposure. Recently, we have shown that posture and the period of time of lying supine can alter both BP and plasma [NO_2_^−^] (Liddle et al. 2018), which emphasizes that posture should be carefully considered when conducting future research in this area.

#### UV-A light reduces ${{\dot{V}}}{\rm O}_{2}$ and RMR

A notable finding in this study is that ${{\dot{V}}}{\rm O}_{2}$ and RMR fell during both light exposures. To our knowledge, this study is the first to explore the effects of UV-A light on resting metabolism in humans. A significant reduction in resting ${{\dot{V}}}{\rm O}_{2}$ was observed despite no elevation in plasma [NO_2_^−^] during UVA10, whereas a trend for a reduction was found in the presence of a significant elevation in plasma [NO_2_^−^] in UVA20. The reduction in ${{\dot{V}}}{\rm O}_{2}$ following UV-A exposure appears to be transient, however, as values returned to baseline 30 min after exposure in both light exposure conditions. While the mechanisms accounting for the reduction in ${{\dot{V}}}{\rm O}_{2}$ could not be ascertained in the present study, we speculate that the complexity of NO conversion and appearance from NO_2_^−^ or other nitrogen oxides following UV-A challenge may have accounted for this finding. We hypothesized that resting ${{\dot{V}}}{\rm O}_{2}$ would be reduced in the presence of elevated NO_2_^−^ as others have shown that NO_2_^−^ inhibited respiration by ~ 60% when applied to primary skeletal myotubes, in vitro (Larsen et al. 2011). Others have demonstrated that increasing plasma [NO_2_^−^] and NO bioavailability via dietary NO_3_^−^ supplementation reduces ${{\dot{V}}}{\rm O}_{2}$ at rest (Larsen et al. 2014; Whitfield et al. 2016). Larsen and colleagues (2014) speculated that the reduction in RMR was most likely due to an NO-mediated inhibition of cytochrome *c* oxidase (Carr and Ferguson 1990) and found that changes in this parameter following dietary NO_3_^−^ were independent of insulin sensitivity and thyroid hormones. However, Whitfield and Colleagues (2016) observed a similar reduction in whole body ${{\dot{V}}}{\rm O}_{2}$ at rest, in the absence of change in skeletal muscle mitochondrial respiration. It is conceivable that the changes in resting ${{\dot{V}}}{\rm O}_{2}$ observed in the present study following UV-A may have occurred via other NO-related mechanisms involving species distinct from those of the canonical NO_3_^−^–NO_2_^−^—NO pathway. It must also be considered that the absence of an elevation in plasma NO_2_^−^ following UVA10 and the presence in UVA20 may be indicative of an NO-independent mechanism.

#### Functional relevance of the findings

Given that NO plays a pivotal role in the regulation of vascular tone (Stamler et al. 1994) and glucose uptake (Balon and Nadler 1997; Bergandi et al. 2003), augmentation of NO bioavailability through environmental exposure to UV-A light has the potential to have a profound impact on human health. The present data suggests that a minimum dose of 20 J cm^2^ of UV-A light is required to elicit a significant increase in plasma NO_2_^−^, a known marker of NO availability. The potential total daily exposure to UV-A light during daylight hours is likely to exceed 20 J cm^2^ in most countries during the spring and summer months (Marionnet et al. 2015). This suggests that habitual exposure to sunlight may complement endogenous NO production and exogenous NO generation from dietary pathways although the aforementioned differences between artificial and natural light should be reemphasized at this stage. Clothing, working patterns, and leisure behaviors are also likely to have a considerable impact on the exposure of skin to UV-A light. Furthermore, in the winter months, countries further from the equator are likely to see daily UV-A exposure fall well below the minimum threshold required to increase NO_2_^−^ (Liu et al. 2014). Indeed, incidences of acute coronary syndrome and stroke are known to be higher in winter (Rosengren et al. 1999; Oberg et al. 2000). Importantly, epidemiological data also suggests that sunlight exposure reduces all-cause and cardiovascular mortality (Yang et al. 2011; Brondum-Jacobsen et al. 2013). There may be value, therefore, in a targeted approach to increase NO availability via the ingestion of NO_3_^−^-rich food and beverages during these periods of reduced UV exposure.

The long term effects of the reduction in resting ${{\dot{V}}}{\rm O}_{2}$ and RMR following UV-A exposure are unclear but there may be positive effects on cellular function and signaling given that an augmented plasma NO_2_^−^ can improve mitochondrial efficiency (Larsen et al. 2011). This may be particularly relevent where tissue oxygen supply is reduced through either clinical (Kenjale et al. 2011) or environmental conditions (Muggeridge et al. 2014). Conversely, a sustained reduction in RMR must be considered as contraindicative for energy balance. For example, Hill et al. (2003) estimated that, on average, the gain of body weight over time was due to a positive energy balance of 15 kcal/day^−1^. This is substantially lower than the reductions in RMR which were induced by exposure to UV-A light in the present study (57–129 kcal/day^−1^). This is of relevance given the composition of UV-A in overall sunlight exposure is approximately 90% although the intensity depends on latitude and seasonal variations in the light/dark cycle (Diffey 2002). These current data would seem to conflict with the suggestion that UV exposure is as a potential intervention for obesity (Geldenhuys et al. 2014; Fleury et al. 2016), potentially mediated via vitamin D or NO effects (Gorman et al. 2017). The present study highlights the extent to which sunlight exposure may have a potentially confounding impact on key markers of cardiometabolic health; data which should be carefully considered in epidemiological research.

#### Limitations

The present study is not without limitations. First, the study lacked ecological validity in the sense that acute exposure to artificial UV-A light in the laboratory tells us little about the relevance of environmental sunlight exposure for habitual vascular homeostasis. Second, while our data suggests there is a minimum dose of UV-A light exposure that is required to elicit meaningful increases in NO bioavailability, the existence of a dose–response relationship cannot be ascertained without more extensive investigations. Although we observed an increase in plasma [NO_2_^−^] following 30 min of simulated sunlight exposure, we did not assess its release from skin stores and are therefore unable to provide a comprehensive assessment of the kinetic changes in NO derivatives and release from the skin. Such data are necessary to truly understand the role of the skin in the regulation of NO bioavailability and to establish whether UV-A exposure may be cardioprotective.

### Conclusions

The present study is the first to determine that at least 20 J cm^2^ of UV-A exposure (~ 30 min of Mediterranean summer sunshine) is required to significantly increase plasma [NO_2_^−^] in healthy human participants. Surprisingly, elevations in plasma NO availability were not met with a significant reduction in BP, which has been consistently observed in previous studies. These contrasting data may relate to experimental differences in UV-A exposure protocols or inter-individual variability in responses between participants. Although BP was not reduced in this study, UV-A exposure in doses of either 10 or 20 J cm^2^ reduced resting ${{\dot{V}}}{\rm O}_{2}$ and RMR. These data provide further evidence to suggest that environmental exposures of sunlight may have a meaningful impact on cardiovascular and metabolic functions.

## Abbreviations

ANOVA: Analysis of variance
BP: Blood pressure
cm: Centimeters
CON: Control
°C: Degrees Celsius
°N: Degrees north
Δ: Delta
DBP: Diastolic BP
HR: Heart rate
IV: Intravenous
J cm^2^: Joules per centimeter squared
Kg: Kilograms
L: Liter
MAP: Mean arterial BP
µL: Microliter
µM: Micromolar
mM: Millimolar
Min: Minutes
nm: Nanometers
nM: Nanomolar
NO_3_^−^: Nitrate
NO: Nitric oxide
NOS: Nitric oxide synthases
NO_2_^−^: Nitrite
*n*: Number
$${{\dot{V}}}{\rm O}_{2}$$: Oxygen utilization
RMR: Resting metabolic rate
NaOH: Sodium hydroxide
SEM: Standard error of the mean
SBP: Systolic BP
RXNO: Total nitrosated products
UV: Ultraviolet
UV-A: Ultraviolet-A
UVR: Ultraviolet radiation
w/v: Weighted volume
XOR: Xanthinine oxioreductases
ZnSO_4_: Zinc sulfate
UVA10: 10 J cm^2^
UVA20: 20 J cm^2^
